# Association of Pyoderma Gangrenosum, Acne, and Suppurative Hidradenitis (PASH) Shares Genetic and Cytokine Profiles With Other Autoinflammatory Diseases: Erratum

**DOI:** 10.1097/01.md.0000464942.01384.16

**Published:** 2015-04-17

In the article “Association of Pyoderma Gangrenosum, Acne, and Suppurative Hidradenitis (PASH) Shares Genetic and Cytokine Profiles With Other Autoinflammatory Diseases”,^1^ which appeared in Volume 93, Issue 27 of *Medicine*, Pier Luigi Meroni was not correctly listed as being affiliated with the IRCCS Istituto Auxologico Italiano, Milano, Italy. The article has since been corrected online.
